# *Solanaceae* Glycoalkaloids Disturb Lipid Metabolism in the *Tenebrio molitor* Beetle

**DOI:** 10.3390/metabo13121179

**Published:** 2023-11-30

**Authors:** Magdalena Joanna Winkiel, Szymon Chowański, Marek Gołębiowski, Sabino Aurelio Bufo, Małgorzata Słocińska

**Affiliations:** 1 Department of Animal Physiology and Developmental Biology, Institute of Experimental Biology, Faculty of Biology, Adam Mickiewicz University in Poznań, ul. Uniwersytetu Poznańskiego 6, 61-614 Poznań, Poland; szyymon@amu.edu.pl (S.C.); malgorzata.slocinska@amu.edu.pl (M.S.); 2 Laboratory of Analysis of Natural Compounds, Department of Environmental Analytics, Faculty of Chemistry, University of Gdańsk, Wita Stwosza 63, 80-308 Gdańsk, Poland; marek.golebiowski@ug.edu.pl; 3 Department of Sciences, University of Basilicata, Via dell’Ateneo Lucano 10, 85100 Potenza, Italy; sabino.bufo@unibas.it; 4 Department of Geography, Environmental Management and Energy Studies, University of Johannesburg, Auckland Park Kingsway Campus, Johannesburg 2092, South Africa

**Keywords:** insect physiology, lipid metabolism, plant secondary metabolites, solanine, chaconine, tomatine, *Solanum lycopersicum*

## Abstract

Glycoalkaloids (GAs) are produced naturally by plants and affect insect survivability and fertility. These compounds can be considered potential bioinsecticides; however, the mechanisms and effects of their action remain undiscovered. As lipids are essential molecules for the proper functioning of an insect organism, this research aimed to determine the effects of GAs on the lipid metabolism of the *Tenebrio molitor* beetle. Solanine, chaconine, tomatine, and tomato leaf extract were applied to larvae by injection at two concentrations, 10^−8^ and 10^−5^ M. Then, the tissue was isolated after 2 and 24 h to determine the levels of free fatty acids, sterols and esters using the GC–MS technique. Moreover, the triacylglyceride level and the activity of the key β-oxidation enzyme, 3-hydroxyacyl-CoA dehydrogenase (HADH), were measured. The results indicate that GAs affect the content and composition of lipid compounds in the beetles’ haemolymph and fat body. The effects depend on the GA concentrations, incubation time, and kind of tissue. Moreover, the tested compounds decrease HADH activity, especially in the fat body, which may affect energy production. To our knowledge, this is the first study concerning lipid metabolism in *T. molitor* after GA application. Our results provide some insights into that topic.

## 1. Introduction

Given the gaps in understanding how GAs influence insect lipid metabolism, this research seeks to provide some new insights into this topic. As lipids are key compounds in all living organisms, we believe that it is an important topic to explore. The insect body requires many appropriate nutrients for proper functioning. Lipid compounds fulfil energy storage (triacylglycerides, TAGs) and cell membrane creation (phospholipids) functions. Cholesterol is responsible for the regulation of plasmalemma fluidity and acts as a precursor for steroid hormone synthesis. Some lipids, such as fatty acids (FA) and phospholipids, can be synthesized by insects in lipogenesis processes, mainly from carbohydrates. Nevertheless, others, such as sterols, must be absorbed from food. The main magazine of lipids in the insect body is the fat body, an organ unique to insects, which performs functions of vertebrate liver and adipose tissue. The fat body is a multifunctional organ constituting the energy metabolism center responsible for nutrient synthesis, storage, and regulation of the endocrine system. Nevertheless, it is also involved in immunity and detoxification processes [1]. Lipids are stored in adipocyte cells primarily as lipid droplets of TAGs, esters of FA with glycerol. TAGs constitute more than 90% of the lipids stored [2]. The fat body is immersed in the haemolymph, which is a tissue analogical to blood in vertebrates. Haemolymph provides great exposure and enables the exchange of metabolites between the tissues. Furthermore, it ensures easy access to nutrients in high energy demand conditions. Diacylglycerides (DAG) and phospholipids, but in smaller amounts also TAGs, FAs, and cholesterol, are transported through the haemolymph in the form of high-density lipoproteins, called lipophorins. Hence, when transport to other tissues/organs (alary muscles, ovary) occurs, the resynthesis of DAG is necessary [1,3]. However, only free FA might be used as a source of energy. Thus, first, they must be released from stored forms (TAG or DAG) by cytosolic lipases. In the next step, they are converted in β-oxidation into acetyl Co-A or propionyl Co-A, NADH, and FADH_2_. One of the enzymes involved in that process is hydroxyacyl-CoA dehydrogenase (HADH), which oxidizes 3-hydroxyacyl-CoA to 3-ketoacyl-CoA with NADH production [4]. Lipid metabolism undergoes strict regulation by the endocrine system, including through neurohormones such as adipokinetic hormones, insulin-like peptides, sulfakinins, and tachykinins [5,6,7,8]. Moreover, the membrane and reserve lipids can be modified by different environmental factors, such as temperature.

In plant-eating insects, many physiological processes can be influenced by various plant-derived compounds. Plants produce a wide range of secondary metabolites (PSMs), such as terpenes, saponins, flavonoids, and alkaloids. Most of these compounds are biologically active substances with antinutritional, deterrent, and even toxic properties against herbivores, bacteria, fungi, and viruses. PSMs might modify neuronal signal transduction, modulate protein synthesis, structure, and activity, interfere with biomembrane components, change their fluidity and permeability, intercalate and alkylate DNA, and induce apoptosis [9]. Some compounds exhibit anticancer activity against different tumours [10]. A wide range of PSM effects can disturb the homeostasis of insect organisms and result in changes in lifetime, survivability, and reproduction, thus affecting the insect population [9,11]. One of the most bioactive groups of PSMs is glycoalkaloids (GAs), which are produced by many plant species, especially *Solanaceae* plants, such as potato (*Solanum tuberosum*), tomato (*Solanum lycopersicum*), and eggplant (*Solanum melongena*). These compounds can alter insect physiology, disturbing metabolism, development, reproduction, or cardiovascular system functioning.

Gas impact insect feeding and body mass gain and have many other effects that were reviewed by Chowanski et al., 2016 [12]. For example, *Solanum* Gas might inactivate acetylcholinesterase (AchE). Molecular docking studies showed that among 25 studied *Solanaceae* alkaloids, solanine had the highest inhibitory activity against AchE in the *Aedes aegypti* mosquito [13]. *Solanaceae* Gas modify heart activity in *Tenebrio molitor* [14] and *Zophobas atratus* beetles [15]. Solanine and chaconine also decreased the cardioinhibitory activity of the calcium channel blocker verapamil in the *T. molitor* beetle [16]. Pure Gas and extracts from the leaves of *S. tuberosum* and *S. lycopersicum* cause developmental abnormalities and reproductive disturbances in *Drosophila melanogaster* [17], whereas pure solanine and chaconine in the diet reduce *Spodoptera exigua* larval growth [18] and exhibit toxicity against the Coleopteran species *Tribolium castaneum*, *Sitophilus oryzae*, and *Trogoderma granarium* [19,20]. Moreover, it was shown that solanine and the potato leaf extract affect the amount of malondialdehyde (MDA), protein carbonyl and glutathione S-transferase activity in *Galleria mellonella* tissues. Thus, they generate oxidative stress and, as a consequence, alter the survivorship, fecundity, and fertility of this insect [21]. It was also shown that *S. nigrum* extract generates changes in the ultrastructure of the midgut and fat body and modifies muscle contractility in the hindgut of *T. molitor*, which can contribute to affected food intake. Furthermore, heterogeneous lipid droplets of irregular shape were observed in the fat body tissue, the most visible in higher extract and pure GA concentrations [22]. Moreover, in *G. mellonella* insects, solasonine and *S. nigrum* extracts change the density of the cytoplasm and the lipid droplet homogeneity in fat body tissue. At the same time, in the midgut, they induce vacuolization and increase the amount of endoplasmic reticulum [23]. Nevertheless, most studies concerning GA activity show various effects of these compounds in insects but usually do not explain their mode of action.

Even less is found in the literature on how GAs affect insect metabolism, with particular emphasis on lipid metabolism. Some studies relate to the lipid profile in insects, but most focus on edible insects’ nutritional values. Additionally, the final effects, such as the survivability of pests treated with GAs, were studied. However, to the best of our knowledge, no studies have focused on the mechanisms and effects of GA action on lipid homeostasis in insects, such as lipid profiles and FA β-oxidation. Thus far, it was shown that the extract of *S. nigrum* at a concentration of 1% (applied to the food) lowered the lipid content in the fat body of *T. molitor* larvae. A similar effect was observed after 0.1% and 10% solasonine treatment. Pure solasonine and *S. nigrum* extract also affected the metabolite content in the haemolymph and fat body in *G. mellonella* [23]. As GAs can increase reactive oxygen species (ROS) concentrations in insects [21], they can also indirectly impair lipid compounds. ROS interfere with membrane lipids, especially polyunsaturated fatty acids (PUFAs). It leads to the peroxidation of lipids and the production of highly toxic electrophilic unsaturated aldehydes, which can react with proteins and nucleic acids, causing damage [24]. Malondialdehyde (MDA) is one of the final products of PUFA peroxidation and a marker of oxidative stress in cells. Solanine increased its content in the midgut and fat body in *G. mellonella* larvae, and the effect depended on the GA concentration in the diet [25]. On the other hand, solanine did not affect superoxide anion production or protein carbonylation in the gut of *Spodoptera littoralis* larvae. This GA also did not change the rate of food passage through the digestive tract or the activity of antioxidant enzymes [26].

As lipid homeostasis is critical for the proper functioning of insect organisms, this research aimed to study the content of lipid compounds and the activity of the β-oxidation enzyme HADH in tissues of *T. molitor* beetles after the application of solanine, chaconine, and tomatine. The extract from tomato leaves was also tested to compare the effects of pure GAs and their mix.

## 2. Materials and Methods

### 2.1. Insects

*T. molitor* beetles were obtained from the colony cultured at the Department of Animal Physiology and Developmental Biology at the Faculty of Biology of the Adam Mickiewicz University in Poznań, Poland under constant temperature (26 ± 0.5 °C), humidity (65 ± 5%) and photoperiod 12:12 h light to dark. The food consisted of oat flakes and fresh carrots. Only feeding larvae from the 15th to 16th instar of approximately 120–140 mg of weight were selected for the experiments.

### 2.2. Compounds and Treatment Procedure

Saline solutions of synthetic GAs solanine (≥95.0%), chaconine (≥95.0%), and tomatine (≥95.0%) (Sigma-Aldrich, Merck, Darmstadt, Germany) were used in experiments at concentrations of 10^−8^ M (dosage range for solanine and chaconine 0.12–0.14 pg/mg body mass, for tomatine 0.15–0.17 pg/mg body mass) and 10^−5^ M (dosage range for solanine and chaconine 0.12–0.14 ng/mg body mass, for tomatine 0.15–0.17 ng/mg body mass). These concentrations were selected based on the literature and our previous studies and they provoke different metabolic and developmental disorders [14,15,16,22,23]. GA extract from tomato leaves was obtained from the research group of Prof. Sabino A. Bufo from Basilicata University in Potenza, Italy, and tested by our group [14,15,17]. Extracts of tomato leaves displayed the presence of the major GAs (2.95 ± 0.25%), tomatine, and two other minor GAs lycotetraose, namely, dehydrotomatine and filotomatine [17]. The extract solutions contained the same concentration of tomatine as the 10^−8^ and 10^−5^ M solutions of this GA, which made it possible to compare the effects of the extract and pure GA. Physiological solution isoosmotic for *T. molitor* was used as a control (NaCl 16 mg/mL, KCl 1.4 mg/mL, CaCl_2_ 1 mg/mL). The tested compounds were administered to larvae by injection using a microsyringe (Hamilton) in a volume of 2 μL, and the final concentration in haemolymph was 10^−9^ and 10^−6^ M. The injection was made on the abdominal side of the larva behind the last pair of legs after 8 min of CO_2_ anaesthesia.

### 2.3. Tissue Isolation

Depending on the experimental variant, tissue isolation was performed 2 or 24 h after GA injection. The tissues were isolated after 8 min of anaesthesia with CO_2_. For analysis, the trophic tissues (haemolymph, gut, and fat body) that play a key role in maintaining metabolic balance as well as in detoxification were used [1,27]. Moreover, haemolymph distributes lipids and applied substances through the whole organism of insects. On the other hand, the fat body is engaged in GA hydrolysis/metabolism, and the gut is responsible for the removal of waste metabolites. Haemolymph was collected using an automatic pipette after cutting the legs of the first pair. After decapitation and cutting off the last segment of the abdomen, the larvae were cut along the dorsal side and then spread on the Petri dish with pins. Afterwards, the fat body and gut were washed with saline, isolated with microsurgical tweezers, and placed in Eppendorf tubes. Additionally, guts were cleaned of food residuals. The isolation was performed on ice to avoid sample degradation. After isolation, the samples were weighed to determine the fresh mass of tissues. Before further preparation, samples were stored at −80 °C. In the experiments, samples pooled from several individuals were used.

### 2.4. Lipid Analysis

#### 2.4.1. Sample Preparation

Gas chromatography–mass spectrometry (GC–MS) was used to measure the levels of lipid compounds in the haemolymph and fat body. For analysis, pooled samples were used with *n* ≥ 15 (haemolymph) or *n* ≥ 10 (fat body), and the analyses were performed in triplicate. Each sample contained a minimum of 120 μL of haemolymph or 160 mg of fat body. After isolation, tissues were transferred into 1.5 mL glass bottles with chloroform and methanol 2:1 (*v*/*v*). The prepared samples were stored at 4 °C until the measurements were taken.

For TAG analysis, pooled samples were used with *n* ≥ 2, and for each experimental variant, four independent replicates were performed. Samples for determining TAG content based on enzymatic reactions were prepared as follows. Fat body tissue was collected from two larvae into an empty tube, while 16 μL of haemolymph was transferred into 150 μL of PBS-Tween 0.05% (PBS: Gibco, Thermo Fisher, Waltham, MA, USA 18912014; Tween: Sigma–Aldrich, Merck, Darmstadt, Germany P1379). Then, the tissue samples were frozen in liquid nitrogen and stored at −80 °C until further steps. After thawing, 300 μL of PBS-Tween 0.05% was added to the fat body samples. Subsequently, the fat body and haemolymph samples were homogenized on ice using a pestle homogenizer (Fisherbrand, Ottawa, ON, Canada) and centrifuged (3000 RPM, 10 min, RT). Then, the supernatant was transferred to a new tube, and 2 μL of solution was taken to determine the soluble protein concentration using a Direct Detect spectrometer (Merck) [28]. Next, after incubation at 70 °C for 10 min to inactivate endogenous enzymes, the samples were centrifuged (10,000 RPM, 5 min, 4 °C), and the supernatant was transferred to new tubes. The samples were frozen in liquid nitrogen and stored at −80 °C until the measurements were carried out.

#### 2.4.2. Lipid Level Determination

Free FAs, sterols, and esters were determined with the GC–MS technique according to the method described previously by Szymczak-Cendlak et al., 2022 [28]. Briefly, lipids were extracted in 30 mL of dichloromethane. The solvent was removed from the samples under a gentle stream of nitrogen. Components of extracts were silylated with 100 μL of a mixture of 99% bis(trimethylsilyl)acetamide and 1% chlorotrimethylsilane at 100 °C for 1 h on the day of analysis. Samples were analyzed using GC–MS on a GC/MS QP2010 SE (Shimadzu, Kyoto, Japan) equipped with a fused silica capillary column Zebron–5, 30 m × 0.25 mm i.d. and with a 0.25 µm thick film. Helium was used as the carrier gas. The ion source was maintained at 220 °C. The injector and transfer line temperatures were kept at 310 °C. Electron-impact ionization (electron energy 70 eV) was used. The column temperature was programmed at 4 °C × min^−1^ from 80 (held for 10 min) to 310 °C, which was held for 10 min.

TAGs were determined spectrophotometrically as described previously by Walkowiak-Nowicka et al., 2023 [29]. The TAG level was measured in undiluted haemolymph samples and fat body samples diluted 10 fold with PBS-Tween 0.05%. Each sample (20 μL) was added to two tubes (A and B). Then, 20 μL of PBS-Tween was added to tube A, and 20 μL of triglyceride reagent (Sigma-Aldrich, Merck, T2449) was transferred to tube B. The samples were incubated at 37 °C for 60 min. After incubation, the samples were centrifuged for 3 min at 10,000 RPM, and 30 μL of each sample was transferred to a clear-bottom 96-well plate, mixed with 100 μL of free glycerol reagent (Sigma-Aldrich, Merck, F6428) and incubated for 10 min at 37 °C. The absorbance was measured at wavelength λ = 540 nm at RT with a Synergy H1 Hybrid MultiMode Microplate Reader (BioTek, Winooski, VT, USA). The TAG level in each sample was measured by subtracting the absorbance of tube A from the absorbance of tube B and calculated based on the curve of the glycerol triolein-equivalent standard (Sigma-Aldrich, Merck, G7793). The TAG level is expressed in µg per 1 mg of total soluble protein level because the protein amount does not change in the samples.

### 2.5. Enzyme Activity Analysis

#### 2.5.1. Sample Preparation

The activity of HADH in the gut and fat body was measured in samples pooled from a minimum of 10 individuals. The assays were prepared in three independent replicates for each experimental variant. Tissues were placed in 250 μL or 500 μL of physiological saline, homogenized for 3 min using a handheld pestle homogenizer (Fisherbrand) and centrifuged (10,000 RPM, 10 min, 4 °C). The supernatant was then transferred to new tubes, and the protein concentration was measured. Total soluble protein concentrations in the gut samples ranged between 9.7 and 18.9 mg/mL, and in fat body samples, they ranged between 13.2 and 29.5 mg/mL. Afterwards, the samples were frozen in liquid nitrogen and stored at −80 °C until the measurements were conducted.

#### 2.5.2. Enzyme Activity Measurements

NADH (7.5 mM; Sigma-Aldrich, Merck, 481913); acetoacetyl-CoA (5.9 mM; Sigma-Aldrich, Merck, A1625); EDTA (34.4 mM; Sigma-Aldrich, Merck, E5134); PBS buffer (pH = 6; Gibco, Thermo Fisher, 18912014); and kojic acid (4 mM; Sigma-Aldrich, Merck, K3125) were used to measure the catalytic activity of HADH. The experiment was performed according to the method of Cheung et al., 2015 [30] with some modifications. The gut samples for measuring HADH activity were undiluted. In contrast, fat body samples were diluted to a total protein concentration of 3.0–4.2 µg/μL with 4 mM kojic acid in PBS buffer. Kojic acid was used as a polyphenol oxidase inhibitor to reduce the interference of the polyphenol oxidase reaction product with the product of the reaction catalysed by HADH. The experiments were based on the spectrophotometric technique using a Synergy H1 Hybrid MultiMode Microplate Reader (BioTek, USA). The proportions of reagents per well were as follows: PBS 125 μL, EDTA 10 μL, NADH 2.5 μL, acetoacetyl-CoA 2.5 μL, and sample 10 μL. Acetoacetyl-CoA was added to the plate just before the measurement. The absorbance was measured at wavelength λ = 340 nm at RT for 15 min (1 min intervals). HADH activity was calculated using the highest absorbance, and the absorbance value was measured 8 min later. Enzyme activity was expressed as mmoles of NADH reduced by HADH during 1 min per mg of total soluble protein.

### 2.6. Statistical Analyses

Statistical analysis of the results and graphs were prepared in GraphPad Prism 8.0.1. The normality was checked with the Shapiro–Wilk test. Next, analysis of variance was performed with ANOVA with Dunnett’s multiple comparisons post hoc tests (normal distribution).

## 3. Results

### 3.1. Level of TAGs

**Figure 1:**
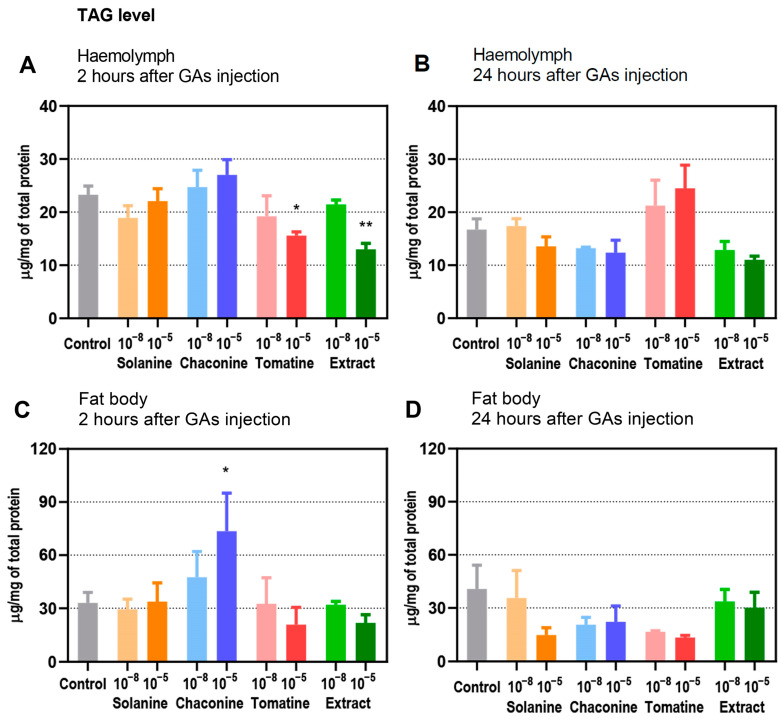
Content of triglycerides (TAGs) in the haemolymph (**A**,**B**) and fat body (**C**,**D**) of *T. molitor* larvae 2 and 24 h after injection with solanine, chaconine, tomatine, extract from tomato leaves and physiological saline as a control. Concentrations of the compounds 10^−8^ M (10^−8^) and 10^−5^ M (10^−5^) are shown on the graphs. The TAG level is expressed in µg per 1 mg of total soluble protein level and shown as the mean with SEM. Pooled samples were used with *n* ≥ 2, and for each experimental variant, four independent replicates were performed. The tested groups were compared to the control (insects injected with physiological saline) using Brown–Forsythe and Welch ANOVA (**A**,**B**) and ordinary one-way ANOVA (**C**,**D**) with Dunnett’s multiple comparisons tests, ** *p* ≤ 0.01, * *p* ≤ 0.05.

The overall amount of TAGs per 1 mg of total protein in control samples or after GA application was higher in the fat body than in the haemolymph at the same incubation time, except for the 24 h experimental variant with tomatine injection (Figure 1).

After 2 h, tomatine (*p* ≤ 0.05) and the extract (*p* ≤ 0.01) at higher tested concentrations decreased TAG levels in the haemolymph (Figure 1A). The level of TAGs in haemolymph as well as in the fat body tended to increase 2 h after 10^−5^ M solanine and 10^−5^ M chaconine administration compared to GAs at a 10^−8^ M concentration (Figure 1A,C). Only chaconine significantly increased TAG level in the fat body compared with the control. This effect was observed for 10^−5^ M GA (Figure 1C). Moreover, the number of TAGs in fat body tissue was higher after 10^−5^ M of chaconine injection than after the application of the same concentration of tomatine (*p* ≤ 0.001) and extract (*p* ≤ 0.001). The opposite situation was observed after the application of tomatine and extract at 10^−5^ M, which caused no statistically significant decrease in fat body tissue, whereas 10^−8^ solutions of GAs evoked only a mild decrease in TAG (Figure 1C).

In the 24 h variant, the TAG level in the haemolymph (Figure 1B) as well as in the fat body (Figure 1D) tended to be lower than that in the control after most GA injections.

### 3.2. Changes in Lipid Composition

**Figure 2:**
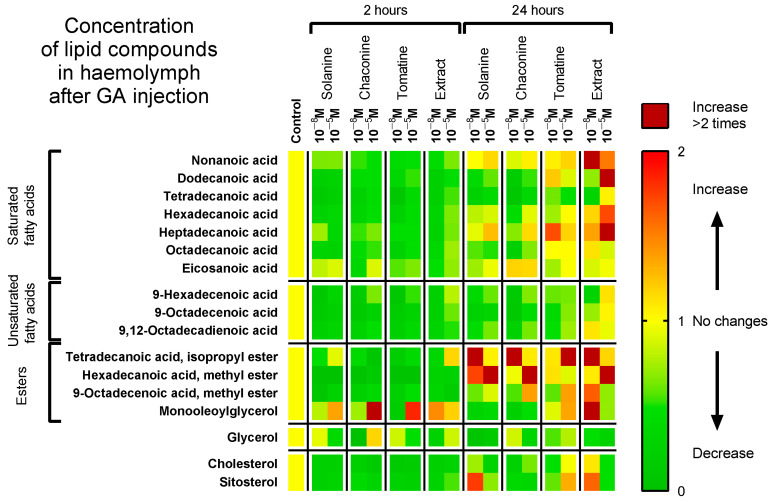
Changes in the composition of lipids identified in the haemolymph of *T. molitor* larvae 2 and 24 h after injection with solanine, chaconine, tomatine, extract from tomato leaves and physiological saline as a control. The graph shows the concentrations of the injected compounds, 10^−8^ M and 10^−5^ M. Changes in the compound levels are marked as yellow—no changes, green—decrease, red—increase, and dark red—increase more than 2 times compared with the control. Pooled samples were used with *n* ≥ 15, and the analysis was performed in triplicate. Two-way ANOVA with Dunnett’s multiple comparisons tests; the lipid levels and *p* values are provided in the Supplementary Materials (Supplementary Table S1).

The obtained data show that the level of lipid compounds in haemolymph 2 h after GA application decreased (Figure 2). The concentration of saturated fatty acids (SFAs) such as myristic, palmitic, and stearic acids and unsaturated fatty acids (UFAs) such as oleic and linoleic acids or cholesterol and sitosterol were significantly lower after GAs compared to the control in all 2 h experimental variants (Supplementary Table S1). For example, the level of myristic acid in the samples from insects treated with solanine 10^−8^ M decreased by 84% (*p* ≤ 0.0001) and by 90% after tomatine 10^−8^ M injections (*p* ≤ 0.0001) compared to the control. After 24 h of incubation, the level of most lipid compounds decreased compared to the control; however, there were fewer changes than 2 h after GA application. Significant differences in palmitic acid concentration could be found, especially in the case of lower GA concentrations. The contents of stearic and myristic acids in the haemolymph were lower mainly after solanine and chaconine injections (both concentrations). Interestingly, the concentration of palmitic acid was observed to exhibit the opposite direction of change after the application of the 10^−5^ M and 10^−8^ M extracts: its level was 1.7 (*p* ≤ 0.0001) and 1.2 (*p* ≤ 0.05) times higher compared to the control. The levels of oleic and linoleic acids after 24 h of incubation decreased significantly, mainly after applying pure GAs at both concentrations (Figure 2). The cholesterol and sitosterol contents were reduced in almost all 24 h experimental variants in comparison to the control; however, 10^−8^ M extract increased their haemolymph levels (*p* ≤ 0.05 and *p* ≤ 0.01, respectively). In the case of most lipid compounds, their level was higher after injection of GAs at 10^−5^ M than after application of GAs at 10^−8^ M. For example, the concentration of linoleic acid in haemolymph 2 h after chaconine 10^−8^ M injection was 2.32 µg/mg and 4.04 µg/mg in the case of chaconine 10^−5^ M application (*p* ≤ 0.0001). Moreover, the content of lipid compounds was usually higher after 24 h of incubation than 2 h after injection. An example is cholesterol, whose concentration 2 h after injection of the tested compounds ranged between 1.58 and 2.96 µg/mg, whereas, after 24 h of incubation, the concentration reached 6.32 µg/mg.

**Figure 3:**
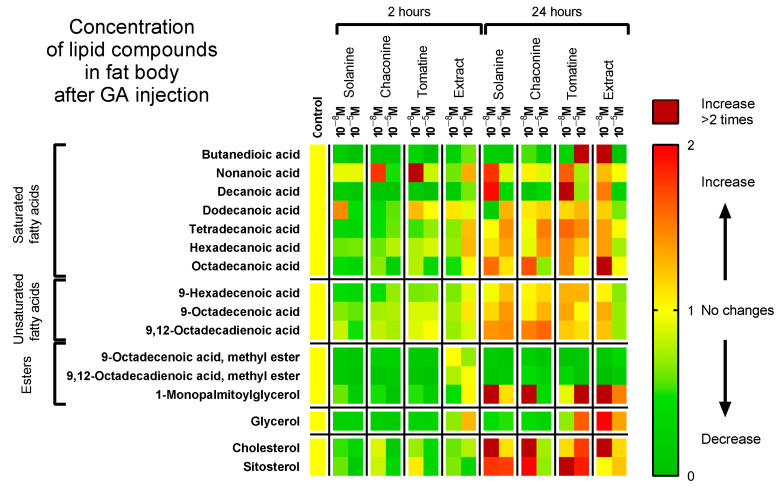
Changes in the composition of lipids identified in the fat body of *T. molitor* larvae 2 and 24 h after injection with solanine, chaconine, tomatine, extract from tomato leaves and physiological saline as a control. The graph shows the concentrations of the injected compounds, 10^−8^ M and 10^−5^ M. Levels of compounds are marked as yellow—no changes, green—decrease, red—increase, and dark red—increase more than 2 times compared with the control. Pooled samples were used with *n* ≥ 10, and the analysis was performed in triplicate. Two-way ANOVA with Dunnett’s multiple comparisons tests; the lipid levels and *p* values are attached in the Supplementary Materials (Supplementary Table S2).

In the case of the fat body, generally, the level of different types of lipids was lower 2 h after GA application and higher after 24 h of incubation compared to the control (Figure 3, Supplementary Table S2). For example, after 2 h of incubation, the concentrations of palmitic, stearic, oleic, and linoleic acids decreased significantly in most experimental variants. Surprisingly, only 10^−5^ M extract increased palmitic acid levels by 29% (*p* ≤ 0.0001) and oleic acid concentrations by 10% (*p* ≤ 0.0001) in the fat body compared with the control. Interestingly, these correlations 24 h after GA injections were reversed: the levels of the mentioned SFAs and UFAs were usually higher, and the extract at a concentration of 10^−5^ M decreased the palmitic acid level by 26% (*p* ≤ 0.0001) and the oleic acid level by 29% (*p* ≤ 0.0001) in this tissue. Similarly, cholesterol levels in the fat body were lower after 2 h of incubation but were usually higher 24 h after GA injections compared with the control. It is worth noting that the sitosterol concentration was decreased by 2 h after the application of all GAs. Nevertheless, it was only 10^−5^ M. Contrary to haemolymph, in the fat body, the level of most lipid compounds was lower after injection of GA at a higher concentration than after application of 10^−8^ M GA. For example, the amount of stearic acid in samples collected 2 h after injections from insects treated with 10^−8^ M tomatine was 12.88 µg/mg, while in those treated with 10^−5^ M tomatine, the amount was 5.53 µg/mg. In most samples, the concentration of lipid compounds was higher after 24 h of incubation than 2 h after injection, so this effect is characteristic of both analyzed tissues. A good illustration is palmitic acid with a concentration of 17.76 µg/mg 24 h after solanine 10^−5^ M injection and 27.76 µg/mg after 2 h of incubation (*p* ≤ 0.0001).

### 3.3. Lipid Content and UFA:SFA Ratio

**Figure 4:**
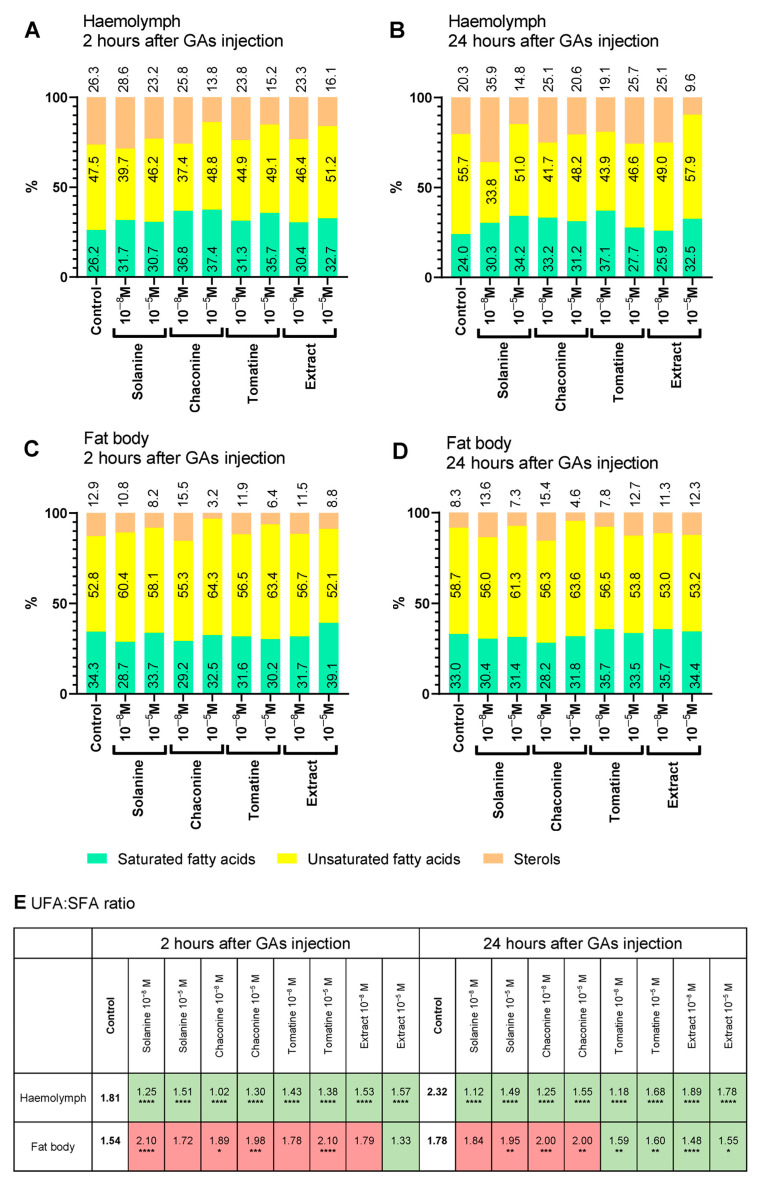
The percentage ratio of saturated fatty acids (SFAs), unsaturated fatty acids (UFA), and sterols in total lipids in haemolymph (**A**,**B**) and fat body (**C**,**D**), and the UFA:SFA ratio (**E**) in *T. molitor* larvae 2 and 24 h after injection with solanine, chaconine, tomatine, extract from tomato leaves and physiological saline as a control. The concentrations of the injected compounds (10^−8^ M and 10^−5^ M) are shown on the graphs. An increase in the UFA:SFA ratio is marked in red, and a decrease is marked in green compared with the control. Pooled samples were used with n ≥ 15 (haemolymph) or n ≥ 10 (fat body), and the analysis was performed in triplicate. The tested groups were compared to the control (insects injected with physiological saline) using two-way ANOVA with Dunnett’s multiple comparisons test, **** *p* ≤ 0.0001, *** *p* ≤ 0.001, ** *p* ≤ 0.01, * *p* ≤ 0.05.

The lipid composition of the haemolymph (Figure 4A,B) and fat body (Figure 4C,D) changed after GA injection. The percentage of SFAs in haemolymph increased compared with the control 2 h after all GA applications (Figure 4A). UFAs were present in higher amounts than in the control after injection of 10^−5^ M GAs, except for solanine. The UFA:SFA ratio was lower than that of the control (1.81) in all experimental variants (Figure 4E). The lowest ratio occurred after chaconine 10^−8^ M (1.02) and solanine 10^−8^ M treatment (1.25). The percentage of sterols in this tissue decreased in samples from insects treated with all the GAs, excluding solanine 10^−8^ M.

Additionally, in all 24 h experimental variants, the percentage of SFAs in haemolymph increased (Figure 4B). Injection of tomatine at both concentrations and the extract at 10^−8^ M did not affect the content of these compounds. On the other hand, after each GA injection, haemolymph from insects contained fewer UFAs (except for extract 10^−5^ M with no impact). Similar to the 2 h incubation, the ratio of UFA:SFA decreased compared with the control (2.32) in all experimental variants, with the lowest value after solanine 10^−8^ M (1.12) and tomatine 10^−8^ M (1.18) treatment (Figure 4E). The content of sterols in haemolymph depended on the GA injected and its concentration. Application of solanine 10^−8^ M, extract 10^−8^ M, and tomatine 10^−5^ M did not affect the sterol percentage in this tissue.

The SFA content was lower in the fat body 2 h after pure GA and extract 10^−8^ M application in comparison to the control (Figure 4C). The opposite situation was observed for UFAs, whose percentage was higher than that in the control in almost all experimental variants. On the other hand, only 10^−5^ M extract caused an increased level of SFAs (from 34.3% in the control to 39.1%) but did not change the content of UFAs. The UFA:SFA ratio was decreased compared with the control (1.54) only after extract 10^−5^ M application (1.33) (Figure 4E). The highest ratio was after solanine 10^−8^ M (2.10) and tomatine 10^−5^ M (2.10) injections. The level of sterols was lower compared to the control in almost all cases—except for chaconine and tomatine at 10^−8^ M, which did not affect their composition.

Twenty-four hours after tomatine and extract injection, SFAs constituted a higher part of the fat body than the control (Figure 4D). Only solanine 10^−5^ M and chaconine 10^−5^ M increased the percentage of UFAs (from 58.7% in the control to 61.3% and 63.6%, respectively). The UFA:SFA ratio decreased compared with the control (1.78) after tomatine and the extract in both concentration injections, with the lowest amount after extract 10^−8^ M application (1.48) (Figure 4E). The highest ratio was calculated after chaconine treatment (ratio 2.00 for both GA concentrations). In most experimental variants, 10^−8^ M GA increased the percentage of sterols in the fat body after 24 h of incubation. Higher concentrations of GA did not impact their levels, except for tomatine.

### 3.4. Enzyme Activity

**Figure 5:**
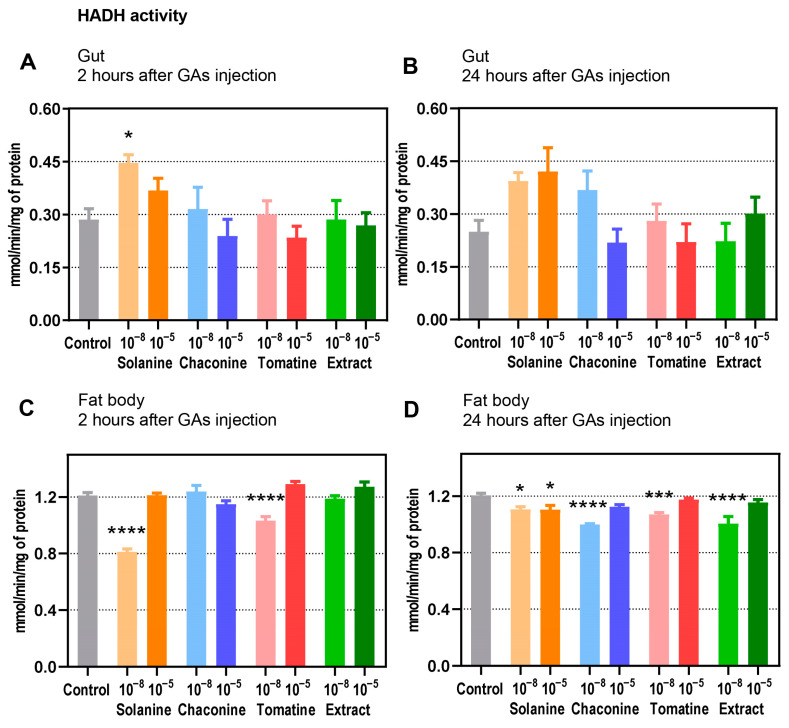
The activity of HADH in the gut (**A**,**B**) and fat body (**C**,**D**) of *T. molitor* larvae 2 and 24 h after injection with solanine, chaconine, tomatine, extract from tomato leaves and physiological saline as a control. The concentrations of the compounds 10^−8^ M (10^−8^) and 10^−5^ M (10^−5^) are shown on the graphs. The HADH activity is expressed as mmol NADH reduced during 1 min per mg of total soluble protein in the sample and is shown as the mean with SEM. Samples were pooled from a minimum of 10 individuals. The assays were prepared in three independent replicates for each experimental variant. The tested groups were compared to the control with ordinary one-way ANOVA, **** *p* ≤ 0.0001, *** *p* ≤ 0.001, * *p* ≤ 0.05.

The activity of HADH in the gut and fat body of *T. molitor* is shown in Figure 5. Generally, the enzyme activity was higher in the fat body than in the gut by almost fivefold.

HADH exhibited 56.3% higher activity in the gut 2 h after solanine application at a concentration of 10^−8^ M when compared with the control (Figure 5A). Moreover, enzyme activity 2 h after application of 10^−8^ M of all GA solutions tended to be higher compared with the 10^−5^ M GAs. Twenty-four hours after the administration of GAs, the tendency of HADH activity was similar at both concentrations, except for solanine and the extract, where higher enzyme activity after 10^−5^ M injections was observed (Figure 5B).

In contrast to the gut, the HADH enzyme in the fat body exhibited a statistically significant drop in activity after solanine 10^−8^ M (32.6% lower activity) and tomatine 10^−8^ M (14,0% lower activity) treatment compared to the control (Figure 5C). Moreover, enzyme activity differed significantly when the same GAs were applied but at different concentrations, for example, after solanine 10^−8^ and 10^−5^ M application (*p* ≤ 0.0001) and after tomatine 10^−8^ and 10^−5^ M treatment (*p* ≤ 0.0001). Different GAs applied at the same concentration also impacted HADH activity differently, such as a 10^−8^ solution of tomatine and extract (*p* ≤ 0.01) or 10^−5^ M chaconine and tomatine (*p* ≤ 0.01). In the 24 h variant, a decrease in HADH activity was observed after applying almost all used GAs at all tested concentrations except for 10^−5^ M chaconine, 10^−5^ M tomatine, and extract 10^−5^ M (Figure 5D). The greatest decrease in the activity compared to the control was observed after chaconine (17.0%), extract (16.4%), and tomatine (11.6%) 10^−8^ M treatments. In 24 h experiments, there were also differences in HADH activity between treatments with the same GAs in different concentrations and between the same concentrations of varying GAs. For example, there was a significantly different effect caused by 10^−8^ M chaconine and solanine, which decreased HADH activity more strongly (*p* ≤ 0.05), and between extract 10^−5^ and 10^−8^ M, where lower HADH activity was observed in this variant (*p* ≤ 0.001).

## 4. Discussion

The purpose of the current study was to determine the impact of GAs on the lipid profile and activity of the β-oxidation enzyme HADH in *T. molitor* larvae. To our knowledge, this is the first study concerning the lipid composition in the *T. molitor* beetle after GA application. We analyzed the concentration of lipid compounds 2 and 24 h after GA injection into the larvae of this insect. Our study found that GAs change the content and composition of many lipid compounds in the haemolymph and fat body of the beetles, depending on the injected substance, incubation time, concentration of the GAs, and type of tissue.

Stored energy is crucial during high energy demand processes, such as growth, reproduction, and flight. Fat reservoirs are essential in holometabolous insects, as they are also used during metamorphosis [5]. TAGs constitute over 90% of lipids stored in fat body tissue [2]. This lipidic content is consistent with our results because TAG levels in the fat body were significantly higher than in haemolymph in almost all experimental variants and samples from insects treated with GAs (Figure 1). Only tomatine and the extract at higher concentrations caused a significant decrease in haemolymph (Figure 1A). At the same time, 10^−5^ M chaconine increased TAG levels in the fat body compared with the control experiment (Figure 1C). The changes were the most evident only 2 h after injection of GAs, which may indicate returning the organism to metabolic balance with time, so the effect of GAs was not observed (Figure 1B,D), although a decreasing trend was still slightly visible. The 10^−5^ M extract lowered the TAG content much more than the 10^−5^ M tomatine extract (Figure 1A). This effect can be explained by the other compounds present in the extract, which can act synergistically with tomatine. These results match previous findings about synergistic effects caused by plant extracts in some Coleoptera species [15,19,20].

As TAG amount is closely related to the fatty acid profile, changes in TAG levels may affect the level of individual FAs. Most of the insects’ energy for physiological processes comes from β-oxidation reactions, where FAs are used as substrates. FAs present in insect tissues usually have 6–20 carbon atom chains. The most common are FAs with 16 and 18 carbon atoms, such as palmitic, stearic, palmitoleic, oleic, and linoleic FAs. In one of the Coleoptera species (*Anthonomus*), palmitic and oleic acids constitute more than half of all FAs [1]. According to the research of [31] in *T. molitor* larvae, the following FAs are present in order of decreasing content: oleic, linoleic, palmitic, myristic, palmitoleic, stearic, linolenic, eicosenoic, γ-linoleic, and vaccenic. Most of them constitute UFAs. The results of our study indicate that the most abundant FAs in haemolymph and fat body tissue were oleic, linoleic, palmitic, stearic, myristic, and palmitoleic acids (decreasing content order). The remaining detected FAs (succinic, pelargonic, capric, lauric, margaric, and arachidic) were present in the larvae at less than 0.5 µg/mg of fresh tissue. Thus, the results obtained in our control experiments seem to be mostly in accordance with the findings of Ravzanaadii et al., 2012 [31]. The exception is, e.g., a higher content of myristic than stearic FA in the cited study. There are several possible explanations for these differences. First, the authors of the research used whole larvae for measurements. Second, the rearing conditions differed slightly. Finally, the diet contained wheat bran, while our larvae were fed oat flakes. Since *T. molitor* beetles were accepted for consumption by the European Union, many studies appeared concerning the relationship between the insect diet and their metabolic profiles to improve the nutritional values [32,33,34]. In addition to its energy storage function, FA plays numerous important roles in the cellular processes of insects [35,36]. They are necessary to produce pheromones, waxes, defensive secretions, and secondary metabolites, which act as sexual attractants, alarm or defensive signals, and influence insect feeding [5,36]. As FAs are cell membrane components, they can affect fluidity. Linoleic and arachidonic acids are involved in synthesizing prostaglandins and other eicosanoids, which are crucial compounds, e.g., in reproduction and the immune response. Cuticular FAs decrease transpiration and desiccation. Furthermore, FAs take part in thermoregulation and camouflage [36,37]. The FA profile is regulated by many factors, such as adipokinetic hormone (AKH) or sulfakinins [38].

As FAs are crucial to maintaining insect homeostasis, compounds that can disturb FA composition and affect insect conditions can be considered potential substances that consequently lead to a reduction in insect populations. Generally, all of the studied GAs lowered the concentration of FA (SFAs and UFAs) in the haemolymph for 24 h (Figure 2) and in the fat body for 2 h (Figure 3). On the other hand, they elevated the FA content in the fat body 24 h after application. This may indicate GA accumulation in that tissue. This can be because of the higher metabolic activity and increased energy demand caused by GA intoxication, as the fat body is the most important site for insect detoxification [39]. Extracts, in some cases, cause different effects than pure GA. For example, they increased the palmitic and oleic acid levels in the fat body after 2 h and decreased 24 h after administration. Additionally, even different concentrations of the extract resulted in other change directions. For example, the concentration of linoleic acid 24 h after extract 10^−8^ M application was higher in the fat body, while when extract 10^−5^ M was used, its level was decreased. A possible explanation of this effect might be that compounds other than tomatine extract, such as dehydrotomatine and filotomatine, affected its activity [17].

When we consider different groups of lipid compounds, the UFA:SFA ratio is an important parameter because it can determine the cell membrane fluidity, permeability, and, consequently, its mode of action. Generally, the membrane is more liquid with a greater content of unsaturated compounds and UFA:SFA ratio [40]. Insects can change this ratio for example during diapause [41,42]. There are many examples of GA effects on cell membranes, including changing the membrane potential and disturbing ion transport from studies of human epithelium, rat cell lines, the gut of mice, rats and hamsters, and frog skin and embryos, as reviewed by Friedman et al. [43,44,45], as well as studies using model cell membrane systems and cancer cell lines [46,47]. Moreover, the length of the acid chain and the number of double bonds in the molecule affect the exact energy yield of the different lipid compounds [48]. In cellular metabolism, SFAs provide more energy than an equivalent amount of UFAs. Our results show that GAs increased the UFA:SFA ratio in the fat body tissue after 2 and 24 h, except for tomatine and the extract after 24 h. Interestingly, in the haemolymph, the UFA:SFA ratio was lower than that in the control in all experimental variants (Figure 4). Thus, the change in the UFA:SFA ratio evoked by GAs indicates that these compounds may affect insect cell membrane functions and energy production by regulating their fluidity. The second component of TAG molecules is glycerol, which builds up other crucial lipid compounds, e.g., phospholipids, and occurs in a free form, mainly in the haemolymph. Glycerol content increased only 24 h after tomatine and extract injections in the fat body tissue. In other experimental variants, the glycerol level was lower than that in the control (Figure 2 and Figure 3).

Cell membrane fluidity can be affected also by sterols. These compounds function as precursors for the synthesis of steroid hormones, too. They are necessary elements for the functioning of all insects, including Coleoptera. They can be derived from their organisms with a diet or be synthesized by microbial symbionts, such as in the *Laspoderma serrlione* beetle. The primary sterol is cholesterol, which builds cell membranes and is a precursor of ecdysteroid hormones. Many insects can convert plant sterols to cholesterol, e.g., sitosterol, campesterol, stigmasterol, and ergosterol [2]. After GA application, sterols exhibited similar changes to FA—their content usually decreased in haemolymph, while increasing in the fat body 24 h after 10^−8^ M GA application (Figure 2, Figure 3 and Figure 4). Thus, as GAs affect the cholesterol content in insect tissues, they can also disturb cell membranes and hormone synthesis [47]. Numerous other PSMs can also affect biomembranes, e.g., saponins, which have an amphiphilic nature and can solubilize cell membranes, increasing their permeability. The lipophilic part of this compound interacts with the bilayer, while the hydrophilic sugar chain acts with glycoproteins and glycolipids, forming pores. Another example is lipophilic terpenes, which bind to cell membranes and disturb membrane proteins, such as ion channels and pumps [49]. Thus, this may also be the case for GA.

As FAs are the main substrates used in energy production, the enzymes that participate in β-oxidation reactions can affect the level of these lipid compounds. One of the key enzymes which are responsible for energy production is HADH. Generally, the activity of this enzyme is much higher in the fat body than in the gut, which is not surprising due to the functioning of this tissue as a metabolic center and lipid store [1]. In the gut, only solanine injection caused higher HADH activity compared to the control (Figure 5A), whereas in the fat body, GA application decreased enzyme activity (Figure 5C,D). Perhaps GAs, like other PMS, can interact with proteins, affecting their function [49]. Contrary to expectations, 10^−8^ M GA decreased the enzyme activity in the fat body after 24 h, while higher concentrations did not affect its activity (Figure 5D). This result is difficult to explain. We suppose that more concentrated GAs may create membrane channels, while the more diluted GA solution affects only the membrane fluidity, which might impact and differentiate the mechanisms of their action.

Taking all of the above aspects together, in the 24 h variant, when we observed increased content of most FAs in the fat body, simultaneously, the concentration of TAGs decreased, and the activity of HADH was lowered. Thus, the increased amount of FA in the fat body might result from increased hydrolysis of TAGs, which causes the release of FA; additionally, because of the lowered activity of HADH, few FAs are oxidized in β-oxidation. This can cause an accumulation of FA in the fat body. However, we also noticed that the FA level increased in the haemolymph which may indicate that FAs are needed in other tissues or organs. One explanation can be the usage of FA for the synthesis of cuticle components or pheromones, which is in accord with studies indicating that alkaloids from *Cynanchum komarovii* increase the relative content of lipids in the cuticle in *S. litura* larvae [50]. However, the total lipid content in *T. castaneum* treated with harmaline decreased [51], while another alkaloid, nicotine, did not affect lipid stores in honeybee pupae [52]. In summary, GAs change the lipid profile in beetle tissues and affect the activity of enzymes crucial for lipid metabolism. Our study has shown that the effects caused by solanine, chaconine, and tomatine depend on their concentration, as well as the time since application and the kind of target tissue. Furthermore, the activity of the extract from tomato leaves differed from that of pure tomato. Changes in lipid metabolism evoked by GAs can lead to disturbances in development, reproduction, and metamorphosis and, consequently, can reduce the pest population. Furthermore, our results provide deeper insight into the effects of GA action, which may allow the design of safe, selective new generation biopesticides to be used in the future as a part of integrated pest management. Further research should focus on other enzymes and lipid metabolites of lipogenesis, lipolysis, oxidation, ketolysis, ketogenesis, lipid oxidation, and peroxidation reactions as well as the metabolism of other crucial compounds, such as carbohydrates, to determine the exact mechanism of GA action. Thus, considerably more work will need to be carried out to determine precise GA activity in insects.

## Data Availability

Data are contained within the article and Supplementary Materials.

## Supplementary Materials

The following supporting information can be downloaded at: https://www.mdpi.com/article/10.3390/metabo13121179/s1, Table S1: Concentration of lipid compounds in the haemolymph of *T. molitor* beetle 2 and 24 hours after glycoalkaloid application. Table S2: Concentration of lipid compounds in the fat body of *T. molitor* beetle 2 and 24 hours after glycoalkaloid application.

## Abbreviations

HADH	3-hydroxyacyl-CoA dehydrogenase
AKH	adipokinetic hormone
DAG	diacylglyceride
FA	fatty acid
GA	glycoalkaloid
MDA	malondialdehyde
MAG	monoacylglyceride
PSM	plant secondary metabolite
PUFA	polyunsaturated fatty acid
SFA	saturated fatty acid
TAG	triacylglyceride
UFA	unsaturated fatty acid
