# Supplementary material for: Solanaceae Glycoalkaloids Disturb Lipid Metabolism in the Tenebrio molitor Beetle
Source: Metabolites. 2023 Nov 30;13(12):1179. doi: 10.3390/metabo13121179 (PMC10744845; doi:10.3390/metabo13121179)
Supplement: Supplementary file 1 [file metabolites-13-01179-s001.zip › metabolites-2689254-supplementary.pdf]

SUPPLEMENTARY MATERIAL

Table S1. Concentration of lipid compounds in the haemolymph of *T. molitor* beetle 2 and 24 hours after glycoalkaloid application. Pooled samples were used with n ≥ 15 and the analysis were performed in triplicate. Two-way ANOVA with Dunnett's multiple comparisons test, α = 0.05.

Compound / Experimental variant		2 h									24 h								
		Control	Solanine		Chaconine		Tomatine		Extract		Control	Solanine		Chaconine		Tomatine		Extract	
			10^{-8} M	10^{-5} M	10^{-8} M	10^{-5} M	10^{-8} M	10^{-5} M	10^{-8} M	10^{-5} M		10^{-8} M	10^{-5} M	10^{-8} M	10^{-5} M	10^{-8} M	10^{-5} M	10^{-8} M	10^{-5} M
**Myristic acid isopropyl ester**	**[µg/mg]**	**0.1**	0.05	0.09	0.04	0.01	0.02	0.04	0.04	0.12	**0.01**	0.08	0.01	0.02	0.01	0.01	0.01	0.02	0.01
	p	-	0.9996	>0.9999	0.9995	0.9966	0.9973	0.9995	0.9995	0.9999	-	0.9995	>0.9999	>0.9999	>0.9999	>0.9999	>0.9999	>0.9999	>0.9999
**Palmitic acid methyl ester**	**[µg/mg]**	**0.13**	0	0	0.01	0.02	0.01	0.01	0.04	0.03	**0.01**	0.01	0.04	0.01	0.04	0.01	0.01	0.01	0.04
	p	-	0.9694	0.9694	0.9808	0.9893	0.9808	0.9808	0.9966	0.993	-	>0.9999	0.9998	>0.9999	0.9998	>0.9999	>0.9999	>0.9999	0.9998
**Oleic acid methyl ester**	**[µg/mg]**	**0.33**	0.03	0.09	0.07	0.12	0.08	0.15	0.1	0.06	**0.09**	0.05	0.08	0.05	0.12	0.05	0.12	0.14	0.06
	p	-	0.3462	0.5942	0.5056	0.7286	0.5496	0.8482	0.6396	0.4629	-	0.9997	>0.9999	0.9997	0.9998	0.9997	0.9998	0.9997	0.9998
**Monooleoylglycerol**	**[µg/mg]**	**0.18**	0.14	0.24	0.13	0.46	0.03	0.32	0.26	0.21	**0.27**	0.08	0.1	0.06	0.12	0.25	0.37	0.59	0.19
	p	-	0.9997	0.9995	0.9996	0.4221	0.9344	0.9542	0.9973	0.9997	-	0.9218	0.9558	0.8755	0.9781	0.9999	0.9972	0.498	0.9994
**Pelargonic acid**	**[µg/mg]**	**0.05**	0.03	0.03	0.03	0.02	0.02	0.02	0.02	0.03	**0.03**	0.03	0.03	0.03	0.03	0.03	0.03	0.06	0.04
	p	-	0.9999	0.9999	0.9999	0.9997	0.9997	0.9997	0.9997	0.9999	-	>0.9999	>0.9999	>0.9999	>0.9999	>0.9999	>0.9999	0.9998	>0.9999
**Lauric acid**	**[µg/mg]**	**0.12**	0.03	0.03	0.05	0.05	0.04	0.06	0.03	0.05	**0.09**	0.04	0.05	0.02	0.05	0.11	0.08	0.06	0.27
	p	-	0.9966	0.9966	0.9994	0.9994	0.9973	0.9995	0.9966	0.9994	-	0.9997	0.9997	0.9995	0.9997	0.9999	>0.9999	0.9998	0.9403
**Myristic acid**	**[µg/mg]**	**1.11**	0.18	0.25	0.27	0.45	0.11	0.43	0.23	0.6	**1.15**	0.31	0.45	0.11	0.41	0.71	0.53	0.37	1.22
	p	-	<0.0001	<0.0001	<0.0001	0.0007	<0.0001	0.0004	<0.0001	0.0166	-	0.0004	0.0048	<0.0001	0.0024	0.1736	0.0175	0.0012	0.9995
**Palmitic acid**	**[µg/mg]**	**6.64**	1.64	2.39	2.14	3.01	1.71	2.75	1.93	4.29	**3.77**	3.01	3.31	1.62	3.48	2.75	3.85	4.41	6.43
	p	-	<0.0001	<0.0001	<0.0001	<0.0001	<0.0001	<0.0001	<0.0001	<0.0001	-	0.0017	0.1403	<0.0001	0.6069	<0.0001	0.9994	0.0129	<0.0001
**Margaric acid**	**[µg/mg]**	**0.05**	0.03	0.02	0.02	0.03	0.02	0.02	0.01	0.03	**0.03**	0.03	0.04	0.02	0.03	0.05	0.03	0.04	0.1
	p	-	0.9999	0.9997	0.9997	0.9999	0.9997	0.9997	0.9997	0.9999	-	>0.9999	>0.9999	>0.9999	>0.9999	0.9999	>0.9999	>0.9999	0.9995
**Stearic acid**	**[µg/mg]**	**4.75**	1.42	1.31	1.93	2.46	1.22	2.14	1.71	3.45	**3.05**	1.75	1.55	0.64	2.02	3.08	3.12	3.42	2.67
	p	-	<0.0001	<0.0001	<0.0001	<0.0001	<0.0001	<0.0001	<0.0001	<0.0001	-	<0.0001	<0.0001	<0.0001	<0.0001	0.9998	0.9995	0.3369	0.3091
**Arachidic acid**	**[µg/mg]**	**0.06**	0.05	0.06	0.02	0.06	0.04	0.04	0.03	0.04	**0.05**	0.04	0.05	0.06	0.06	0.04	0.05	0.05	0.05
	p	-	>0.9999	>0.9999	0.9997	>0.9999	0.9999	0.9999	0.9997	0.9999	-	>0.9999	>0.9999	>0.9999	>0.9999	>0.9999	>0.9999	>0.9999	>0.9999
**Palmitoleic acid**	**[µg/mg]**	**0.45**	0.05	0.14	0.08	0.29	0.05	0.23	0.09	0.35	**0.32**	0.13	0.19	0.05	0.21	0.19	0.2	0.09	0.36
	p	-	0.101	0.3118	0.1524	0.91	0.101	0.6845	0.1734	0.993	-	0.9218	0.991	0.6809	0.9966	0.991	0.9934	0.8184	0.9997
**Linoleic acid**	**[µg/mg]**	**8.98**	2.05	2.8	2.32	4.04	2.03	3.41	2.73	4.61	**6.48**	2.65	4.07	1.62	4.1	3.13	4.65	7.19	6.08
	p	-	<0.0001	<0.0001	<0.0001	<0.0001	<0.0001	<0.0001	<0.0001	<0.0001	-	<0.0001	<0.0001	<0.0001	<0.0001	<0.0001	<0.0001	0.0041	0.2579
**Oleic acid**	**[µg/mg]**	**13.74**	2.12	3.22	2.14	3.59	2.46	3.87	3.24	8.35	**12.12**	3.03	3.91	1.46	5.08	4.69	8.07	8.59	12.77
	p	-	<0.0001	<0.0001	<0.0001	<0.0001	<0.0001	<0.0001	<0.0001	<0.0001	-	<0.0001	<0.0001	<0.0001	<0.0001	<0.0001	<0.0001	<0.0001	0.011
**Glycerol**	**[µg/mg]**	**1.08**	1	0.42	0.03	1.26	0.91	0.49	0.27	0.91	**1.29**	0.07	0.17	1.11	0.42	0.72	1	0.62	0.36
	p	-	0.9973	0.0007	<0.0001	0.8482	0.8811	0.0033	<0.0001	0.8811	-	<0.0001	<0.0001	0.9403	0.0002	0.036	0.6069	0.008	<0.0001
**Cholesterol**	**[µg/mg]**	**10.58**	2.53	2.43	2.28	1.58	1.84	1.86	2.43	2.96	**5.76**	4.2	1.62	1.46	3.64	2.82	5.63	6.32	2.66
	**p**	-	<0.0001	<0.0001	<0.0001	<0.0001	<0.0001	<0.0001	<0.0001	<0.0001	-	<0.0001	<0.0001	<0.0001	<0.0001	<0.0001	0.991	0.0413	<0.0001
**Sitosterol**	**[µg/mg]**	**2.22**	0.51	0.65	0.86	0.66	0.56	0.47	0.61	1.23	**1.12**	1.96	0.75	0.42	0.37	0.66	1.49	1.8	0.52
	**p**	-	<0.0001	<0.0001	<0.0001	<0.0001	<0.0001	<0.0001	<0.0001	<0.0001	-	0.0004	0.3369	0.0048	0.002	0.1403	0.3369	0.0068	0.0235

Table S2. Concentration of lipid compounds in the fat body of *T. molitor* beetle 2 and 24 hours after glycoalkaloid application. Pooled samples were used with n ≥ 10 and the analysis were performed in triplicate. Two-way ANOVA with Dunnett's multiple comparisons test, α = 0.05.

**Compound / Experimental variant**		**2 h**									**24 h**								
		**Control**	**Solanine**		**Chaconine**		**Tomatine**		**Extract**		**Control**	**Solanine**		**Chaconine**		**Tomatine**		**Extract**	
			**10^{-8} M**	**10^{-5} M**	**10^{-8} M**	**10^{-5} M**	**10^{-8} M**	**10^{-5} M**	**10^{-8} M**	**10^{-5} M**		**10^{-8} M**	**10^{-5} M**	**10^{-8} M**	**10^{-5} M**	**10^{-8} M**	**10^{-5} M**	**10^{-8} M**	**10^{-5} M**
**Linoleic acid methyl ester**	**[µg/mg]**	**0.47**	0.06	0.02	0.03	0.08	0.04	0.02	0.36	0.46	**0.46**	0.05	0.07	0.2	0.11	0.16	0.02	0	0.07
	p	-	0.9836	0.9694	0.9733	0.9878	0.9778	0.9712	0.9997	>0.9999	-	0.8663	0.8888	0.9886	0.9369	0.9703	0.8322	0.7997	0.8888
**Oleic acid methyl ester**	**[µg/mg]**	**0.94**	0.12	0.08	0.24	0.24	0.13	0.15	0.94	0.65	**1.09**	0.2	0.07	0.24	0.07	0.1	0.09	0.13	0.35
	p	-	0.6206	0.573	0.7744	0.7685	0.6374	0.6591	>0.9999	0.9971	-	0.1424	0.0623	0.1755	0.0636	0.0767	0.0697	0.0937	0.2981
**Glyceryl palmitate**	**[µg/mg]**	**0.49**	0.3	0.08	0.24	0.03	0.25	0.09	0.22	0.5	**0.07**	0.34	0.08	0.16	0.03	0.07	0.6	0.15	0.11
	p	-	0.9995	0.9839	0.9994	0.9658	0.9994	0.987	0.9975	>0.9999	-	0.9867	>0.9999	0.9997	0.9999	>0.9999	0.6654	0.9997	0.9999
**Succinic acid**	**[µg/mg]**	**0.38**	0.08	0.01	0.03	0.03	0.12	0	0.1	0.23	**0.12**	0.03	0.02	0.07	0.02	0.04	0.27	0.25	0
	p	-	0.9969	0.9911	0.993	0.9931	0.9976	0.9898	0.9972	0.9996	-	0.9997	0.9996	0.9998	0.9997	0.9997	0.9994	0.9995	0.9996
**Pelargonic acid**	**[µg/mg]**	**0.21**	0.19	0.19	0.37	0.1	0.48	0.17	0.13	0.27	**0.19**	0.34	0.17	0.2	0.17	0.31	0.14	0.24	0.19
	p	-	>0.9999	>0.9999	0.9996	0.9997	0.9974	>0.9999	0.9998	0.9999	-	0.9994	>0.9999	>0.9999	>0.9999	0.9996	0.9998	0.9998	>0.9999
**Capric acid**	**[µg/mg]**	**0.11**	0.02	0.01	0.01	0.01	0.02	0	0.02	0.07	**0.03**	0.06	0.01	0	0.01	0.08	0.02	0.04	0.01
	p	-	0.9998	0.9997	0.9997	0.9997	0.9998	0.9997	0.9998	>0.9999	-	>0.9999	>0.9999	>0.9999	>0.9999	0.9998	>0.9999	>0.9999	>0.9999
**Lauric acid**	**[µg/mg]**	**0.22**	0.33	0.1	0.09	0.13	0.28	0.22	0.24	0.2	**0.21**	0.05	0.28	0.23	0.25	0.25	0.27	0.25	0.14
	p	-	0.9997	0.9997	0.9997	0.9998	0.9999	>0.9999	>0.9999	>0.9999	-	0.9993	0.9998	>0.9999	0.9999	0.9999	0.9998	0.9999	0.9997
**Palmitic acid**	**[µg/mg]**	**28.31**	16.8	17.76	17.2	22.19	21.86	24.7	19.93	36.38	**20.51**	22.99	27.76	18.79	29.16	29.84	24.96	28.17	15.18
	p	-	<0.0001	<0.0001	<0.0001	<0.0001	<0.0001	<0.0001	<0.0001	<0.0001	-	<0.0001	<0.0001	0.0001	<0.0001	<0.0001	<0.0001	<0.0001	<0.0001
**Stearic acid**	**[µg/mg]**	**16.64**	7.26	5.13	11.14	4.28	12.88	5.53	8.17	16.1	**6.78**	10.61	7.47	11.44	4.57	9.79	6.41	14.58	6.59
	p	-	<0.0001	<0.0001	<0.0001	<0.0001	<0.0001	<0.0001	<0.0001	0.9259	-	<0.0001	0.3784	<0.0001	<0.0001	<0.0001	0.9192	<0.0001	0.9973
**Myristic acid**	**[µg/mg]**	**3.57**	1.25	1.09	1.63	2.13	1.88	2.43	2.38	4.47	**2.21**	2.17	3.2	2.08	3.35	3.52	3.22	3.05	2.26
	p	-	0.0007	0.0002	0.0075	0.0889	0.0284	0.2721	0.2271	0.5164	-	0.9999	0.0787	0.9995	0.0281	0.0072	0.0675	0.1832	0.9999
**Palmitoleic acid**	**[µg/mg]**	**1.33**	0.56	0.48	0.62	0.92	0.83	0.87	0.84	1.24	**0.86**	0.83	1.08	0.89	0.99	1.13	1.11	0.89	0.57
	p	-	0.6948	0.5832	0.7625	0.9851	0.951	0.9697	0.957	0.9998	-	>0.9999	0.9966	>0.9999	0.9995	0.988	0.9913	>0.9999	0.976
**Linoleic acid**	**[µg/mg]**	**28.49**	23.67	14	23.14	20.96	25.66	28.02	20.18	24.94	**19.47**	27.69	28.21	29.28	31.09	23.43	21.97	24.04	13.7
	p	-	<0.0001	<0.0001	<0.0001	<0.0001	<0.0001	0.9607	<0.0001	<0.0001	-	<0.0001	<0.0001	<0.0001	<0.0001	<0.0001	<0.0001	<0.0001	<0.0001
**Oleic acid**	**[µg/mg]**	**46.24**	30.31	27.33	33.93	35.21	40.44	40.48	34.29	50.69	**33.14**	38.15	46.65	35.34	42.92	44.93	33.52	44.14	23.42
	p	-	<0.0001	<0.0001	<0.0001	<0.0001	<0.0001	<0.0001	<0.0001	<0.0001	-	<0.0001	<0.0001	<0.0001	<0.0001	<0.0001	0.9035	<0.0001	<0.0001
**Glycerol**	**[µg/mg]**	**4.03**	1	0.96	0.86	0.67	1.25	1.2	2.71	5.17	**2.68**	1.23	1.36	1.13	0.77	1.84	4.38	5.3	3.65
	p	-	<0.0001	<0.0001	<0.0001	<0.0001	<0.0001	<0.0001	0.1405	0.2679	-	0.0018	0.0063	0.0007	<0.0001	0.1765	0.0002	<0.0001	0.0849
**Cholesterol**	**[µg/mg]**	**15.62**	8.07	5.55	13.75	1.98	11	6.01	9.41	12.12	**6.48**	14.26	7.2	15.86	4.73	7.39	11.42	13.72	7.45
	p	-	<0.0001	<0.0001	0.0107	<0.0001	<0.0001	<0.0001	<0.0001	<0.0001	-	<0.0001	0.3207	<0.0001	<0.0001	0.1191	<0.0001	<0.0001	0.0875
**Sitosterol**	**[µg/mg]**	**2.89**	1.72	0.34	2.41	0.89	3.11	0.97	1.85	0.89	**1.06**	1.88	1.85	2.1	0.74	2.2	1.98	1.04	1.29
	p	-	0.2388	0.0001	0.9589	0.0054	0.9994	0.0086	0.3632	0.0052	-	0.2003	0.2356	0.0546	0.9599	0.0261	0.113	>0.9999	0.9933
